# Thrombophilia screening revisited: an issue of personalized medicine

**DOI:** 10.1007/s11239-020-02090-y

**Published:** 2020-04-04

**Authors:** Giuseppe Colucci, Dimitrios A. Tsakiris

**Affiliations:** 1 Faculty of Medicine, University of Basel, Basel, Switzerland; 2 Service of Hematology, Clinica Luganese Moncucco, Via Moncucco 10, 6900 Lugano, Switzerland; 3 Diagnostic Hematology, Department of Hematology, University Hospital Basel, Basel, Switzerland

**Keywords:** Venous thromboembolism, Thrombophilia screening, Genetic thrombophilia

## Abstract

Clinical thrombophilia is the consequence of multiple gene and/or environment interactions. Thrombophilia screening requires a targeted patient with specific indication, in which a finding would have implications. Carrying out a thrombophilia examination in the physician’s practice is often a cause of uncertainty and concern. The concerns begin in choosing the right patient to be examined, are associated with the time of investigation, with the choice of analysis, the test-material and with the correct interpretation of the results. Difficulties, which can influence the results, can occur with both organization and blood sampling. As common for any analysis, pre-analytical, analytical and post-analytical factors should be considered, as well as the possibility of false positive or false negative results. Finally, recommendation of correct therapeutic and prophylactic measures for the patient and his relatives is an additional focus. In this article we want to provide—on the basis of the evidence and personal experience—the theory of thrombophilia-investigation, the indications for testing, as well as practical recommendations for treatment options.

## Highlights

- Thrombophilia screening should be a global, comprehensive, personalized evaluation of the patient’s pro-thrombotic state.
- Global thrombophilia evaluation is indicated in all patients with thromboembolism, whereas thrombophilia-specific laboratory screening only in selected cases.
- Thrombophilia investigation should not be performed just for defining the duration of anticoagulation, but it helps in estimating the individual recurrence risk for thrombotic disease, the need for thrombotic prophylaxis or for the decision to prolong anticoagulation therapy.
- Genetic causes of thrombophilia are significant risk factors for a first thromboembolic event but they do not influence decisively recurrent thrombotic risk.
- Although single genetic thrombophilia is mostly kept in balance in children and young adults, it can cause serious thrombotic disease in adults, as soon as acquired risk factors are additionally prevalent (gene-environment interaction) or if multiple deviations in thrombophilia genes co-exist (gene-gene interaction).

## Introduction

Thrombophilia—θρομβοφιλία—originating from Greek is a term meaning both affinity (-philia: φιλία) and blood clot (θρομβο). Thrombophilia indicates an increased tendency to form pathological intravascular venous or arterial thrombosis, mainly as a consequence of interaction of multiple inherited and/or acquired predisposing factors [1]. The coagulation system—usually in a balance between pro- and anticoagulant influences—shifts towards a pro-thrombotic state which may become clinically manifest as a thromboembolic disease [2]. The clinical complexity to understand the in-vivo mechanism(s) shifting the coagulation into a pro-thrombotic state—i.e. a state with an excessive thrombin generation—is the result not only of numerous coagulation factors and their interactions, but also the result of their dynamic interactions with blood vessels, endothelial cells, platelets and other cells in the circulation. Venous stasis from impaired blood circulation was already described from Rudolf Virchow in 1856 as one of the main etiologic factors for venous thrombosis [3]. Although the role of venous stasis was accepted, the link between stasis and thrombosis for example after a long flight or immobilization remained for long time elusive [4–6]. A reduction of the fibrinolytic capacity is conceivable [7] but evidence in the human model is lacking [8]. The interactions between coagulation and other humoral systems such as complement [9] and immune system [10] are ongoing and complex. The risk of thrombosis increases ultimately with age as a consequence of punctual and constant influencing factors [11]. Venous thromboembolism (VTE) is therefore considered a multifactorial disease [12] and the final clinical sign of interaction of single or multiple genetic, epigenetic and/or acquired predisposing factors [1, 13]. Therefore, focusing on and screening for hereditary thrombophilia should represent a comprehensive evaluation of the patient’s pro-thrombotic state and not a purely laboratory testing.

Despite the association of genetic risk factors with VTE, screening for inherited thrombophilia has not shown a direct clinical benefit in the management of these patients [14]. The lack of preventive and therapeutic consequences after the first thrombotic event reduced the indications for thrombophilia screening [15, 16]. Indeed, not testing blindly for thrombophilia in patients with VTE is on the Choosing Wisely list endorsed by many scientific societies [17–19]. But, it seems that the books are still not closed. In the era of big-data acquisition and genome-wide association studies (GWAS), it might be that newer aspects concerning risk evaluation for thrombosis come into consideration, based on the influence of newly identified genetic variations linked to thrombosis.

In this contest, in order to act against an indiscriminate, universal, population screening and to limit medical expenses we suggest to first carefully select the patient. In this review, based on evidence and personal clinical experience, we propose arguments favoring an individual decision-making regarding thrombophilia screening only in selected patients with VTE.

## Venous thromboembolism

VTE includes the deep venous thrombosis (DVT) in typical or atypical localization and pulmonary embolism (PE). VTE may be divided between provoked (secondary) and unprovoked (idiopathic) (Table 1). Risk factors for provoked VTE are major, minor, transitory (reversible) or persistent (irreversible) (Table 2). Differentiation between idiopathic and secondary VTE is important because it affects the decision on the duration of antithrombotic therapy [20].

**Table 1:** Risk factors for venous thromboembolism

Provoking risk factors	Non-provoking risk factors	Genetic risk factors
Cancer	Age > 60 years	Antithrombin-deficiency
Surgery	Sex	Antithrombin-resistance
Trauma	Ethnicity	Protein C deficiency
Acute infection	Oral contraceptive	Protein S deficiency
Immobilization	Hormone therapy	Factor V-Leiden (G1691A)
Pregnancy	BMI	Factor II-Mutation (G20210A)
Post-partum period		Elevated FVIII level
Long distance travel		Dysfibrinogenemia
Hospitalization		Blood group Non-O
Catheterization		Loci for VTE susceptibility: *TSPAN15, SLC44A2*

Adapted from [22] and [83]

**Table 2:** Thrombophilia: risk factors graded as major, minor, persistent, transient

Major persistent	Major transient
Male sex	Surgery
Age > 65 years	Trauma
Active cancer	Cesarean section
Myeloproliferative neoplasm	Pregnancy—Puerperium
Antiphospholipid syndrome	Severe infection
Behçet disease—Hughes–Stovin syndrome	Nephrotic syndrome
Cushing syndrome	
Paroxysmal nocturnal hemoglobinuria (PNH)	
Klinefelter syndrome	
Sickle cell disease	
Some forms of inherited thrombophilia	

Minor persistent	Minor transient
Some forms of inherited thrombophilia	Smoking
Non-O blood group	Dehydration
BMI > 30 kg/m2	Treatment with synthetic estrogens
Post thrombotic syndrome	Varicosis
Chronic bowel inflammatory disease	Immobilisation—Flight > 4 h
Lower extremity paralysis or paresis	Intermittent chemotherapy
Congestive heart failure	Testosterone therapy
Depression	
Lupus anticoagulants	
Calculated creatinine clearance < 50 mL per minutes	

Adapted from [49]

## Inherited thrombophilia

Genetic risk factors for VTE are deficiencies of natural anticoagulant proteins (antithrombin deficiency [21], protein C deficiency [22], protein S deficiency [23]), genetic dysfibrinogenemia [24], hyperhomocysteinemia [25] or mutation of factor II (*F2*, G20210A mutation [26]) or factor V-Leiden (*F5*, G1691A mutation [27]). GWAS revealed several additional genetic polymorphisms with borderline but measurable statistical association with VTE [13, 28]. The non O-blood group status is in this sense the most common mild predisposition [29–31]. Two VTE associated loci, *TSPAN15* and *SLC44A2*, increase also slightly the odds ratio for VTE (1.31 for *TSPAN15* and 1.21 for *SLC44A2*, respectively) [32]. A comprehensive review about the genetics of VTE describing the allele prevalence and influence on VTE of various genetic variances was recently published [33]. The ThromboGenomics group in the United Kingdom using next generation sequencing and a high-throughput screening panel for genetic analysis of patients with coagulation, platelet or thrombotic disorders was able to identify a genetic diagnosis in 48.9% of patients with thrombotic disease [34].

Hereditary thrombophilia predisposes to an imbalance of the coagulation mechanisms. The cumulative prevalence of hereditary thrombophilic defects in the general population is not rare [35, 36] (Table 3). Still, these defects should be excluded only in patients with a specific indication. Inherited thrombophilia in combination with acquired thrombophilic risk factors—which may be transient or persistent [37]—may lead to VTE at young age. Inherited thrombophilia has a different impact on the relative risk of first VTE or recurrence: hereditary thrombophilia increases the relative risk of the first thrombosis, while the risk of recurrence is marginally but still not negligibly affected [38] (Table 4).

**Table 3:** Prevalence of thrombophilic defects in the general population and patients with VTE

	Prevalence in the general population (%)	Prevalence in VTE cohort (%)	Annual VTE Risk (%/y)
Antithrombin deficiency	0.02	0.5	1.1
Protein C deficiency	0.15	6	0.7
Protein S deficiency	0.1	2	0.3
FV Leiden heterozygous	5	16	0.5
FV Leiden homozygous	0.004	0.01	1.3
FII G20210A heterozygous	2	7	0.4
FII G20210A homozygous	0.1	2	1.1
FV Leiden/FII heterozygous	0.1	3	0.5

**Table 4:** Relative risk for first and recurrent thromboembolic events in inherited Thrombophilia [38]

Relative Risk	First VTE	Recurrent VTE
Factor V Leiden		
Heterozygous	4.9–9.7	1.3
Homozygous	40–80	–
Factor II-Mutation	1.9–3.8	1.4
Antithrombin deficiency	5–8	0.5
Protein C deficiency	5–8	2.5
Protein S deficiency	1.7–8	2.5
Dysfibrinogenemia	–	
Hyperhomocysteinemia	–	
Non-O blood type	2.5	

## Inherited thrombophilia screening in children

The risk of VTE in children is low with two peaks in neonates and adolescents [39]. Risk factors for VTE in these two categories of patients are sepsis, dehydration, congenital heart failure, congenital anomalies of vena cava inferior and the use of catheter interventions [40]. In addition, cancer, polychemotherapy, immobilization after surgery or plaster casts, obesity, rheumatic disease, infection and use of oral contraceptive pills in girls were the leading triggers in adolescents [41]. Although one or more of these risk factors are often present in the majority of children with VTE, inherited thrombophilia is significantly associated with the first VTE [41, 42]. Furthermore, a significant association with recurrent VTE in children was found for protein C, protein S, and antithrombin deficiency; the factor II mutation and the combination of two or more genetic traits [41]. In neonates with purpura fulminans, skin necrosis or idiopathic VTE, as well as adolescents with idiopathic VTE, thrombophilia screening is strongly recommended [43].

## Thrombophilia screening in adult patients

**Fig. 1:**
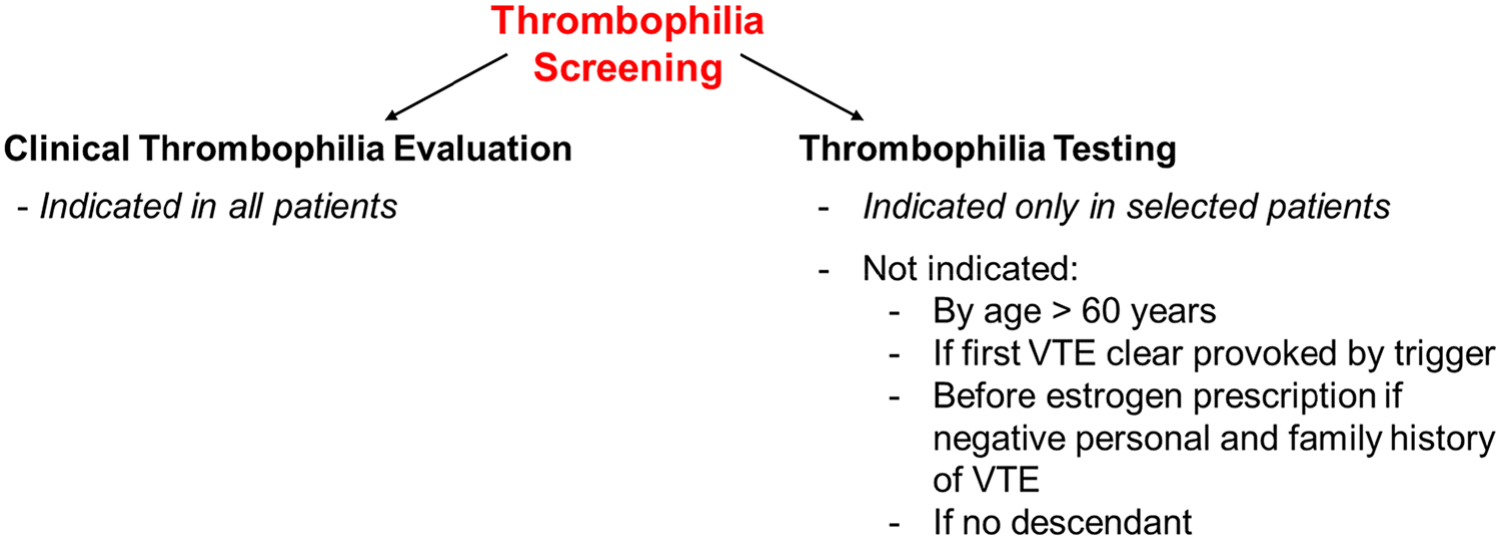
Differentiation between clinical thrombophilia evaluation and thrombophilia testing

Thrombophilia screening—generally performed after a thromboembolic event—is not only a laboratory test for inherited diseases. Thrombophilia screening in adults should be a comprehensive evaluation of the patient’s pro-thrombotic state, allowing to determine the etiology of VTE, to estimate the risk of recurrence, to recommend therapy or prophylactic measures for patient or descendants. These objectives cannot be achieved by purely performing laboratory tests. The clinical evaluation of thrombophilia is based on personal and family history, clinical examination and basic laboratory diagnostics. It is a physician’s task to advance step by step in the evaluation and then to decide if indicated to perform—or not—genetic tests. We suggest to split-up the thrombophilia screening into clinical thrombophilia evaluation and laboratory testing, in particular genetic (Fig. 1).

### Clinical thrombophilia evaluation

All patients with first VTE—i.e. Index patient—should be evaluated clinically for thrombophilia. This starts with a careful personal and family history for venous (or arterial) thrombosis, past clinical history, associated diseases and their prognosis, evaluation of acquired risk factors for VTE and the therapy’s adverse effects. Full physical examination is indicated with particular attention to habitus, skin, cardiovascular i.e. peripheral venous, arterial and lymphatic, lung, abdominal, and neurological systems. Based on information such as age, thrombus localization, i.e. arterial, venous or paradoxical embolism and acquired predisposing risk factors the treating physician decides on the next steps. A basic laboratory test is indicated in all patients (Table 5). An important issue at this point is to carefully select the patient who should be screened with supplementary thrombophilia-specific laboratory tests.

**Table 5:** Basic laboratory tests at the initial examination of thromboembolism

Coagulation	Prothrombin time (PT)
	Activated partial thromboplastin time (aPTT)
	D-Dimer
	Fibrinogen (functional assay) Factor VIII level
	Lupus anticoagulants, anti-cardiolipin, anti-β2 glycoprotein I antibodies
Hematology	Complete blood count
Chemistry	Kidney, liver, infection parameters, lactate dehydrogenase (LDH)

Clinical VTE is multifactorial and the sum of gene–gene and/or gene-environment interactions which lead to overcome a threshold and trigger the event [44]. In this framework of predisposing factors inherited thrombophilia interacts dynamically with environmental factors, which are partly modifiable. Therefore, the single contribution and finally the risk of VTE may change over time [12]. Acquired predisposing factors for VTE—that may be classified in major/minor and persistent/transient (Table 2)—play a different role in the presence of an inherited thrombophilia. Indeed, a minor risk factor may be sufficient to trigger an episode of VTE in a young patient typically in association with a genetic risk factor. On the other hand, most patients over 60 years may develop a VTE in the presence of one or more acquired risk factors, even without an inherited thrombophilia. All predisposing factors that—alone or in combination—increase the risk of VTE should be screened or excluded (Table 2). The combination of two or more factors increases the risk of thrombosis multiplicatively.

In the case of a patient aged > 60 years and/or one or more robust causes of acquired thrombophilia the physician should omit thrombophilia testing, particularly genetic tests. It is important to determine if VTE was triggered by a strong risk factor, i.e. antiphospholipid syndrome, malignancy and so forth. Age and acquired thrombophilia are the most important factors influencing the decision to exclude or not an inherited thrombophilia, including genetic screening.

### Personalized thrombophilia evaluation

Personalized thrombophilia evaluation begins with consideration of patient’s characteristic. Starting with age, i.e > 60 years, 50–60 years or < 50 years and sex the clinician may take a first decision. Because age is a major risk factor for VTE, frequently associated with other persistent or transient risk factors (Table 2), the probability of an underlying genetic cause is very low. Therefore, in patients over 60 years genetic screening should not be performed because not indicated (Table 7).

In patients younger than 60 years, risk factors for VTE should be excluded: in the presence of one major or multiple minor risk factors, no other diagnostic procedures are indicated.

In patients younger than 50 years without apparent risk factors, additionally cancer, PNH or an autoimmune disease should be considered [45]. VTE can be the first presentation of malignancy and symptomatic deep-vein thrombosis is associated with a risk of subsequent overt malignant disease [46]. We suggest personal, familial history, physical examination and starting from clinical symptoms an age- and gender-specific screening [47]. We suggest a limited and against an extended cancer screening [48].

In younger patients, especially under 40 years, additional risk factors like illicit drug consumption or therapy (including cocaine, estrogens, testosterone, erythopoietin) should be considered and excluded. Rare diseases (Klinefelter syndrome, Behcet disease, Hughes–Stovin syndrome, antiphospholipid syndrome, cancer and hematological diseases (i.e. sickle cell disease or neoplasm including leukemia) should be excluded based on clinical probabillity and first laboratory screening (Table 5). It should be kept in mind that a VTE (or an arterial TE by paradoxical embolism) can be the first manifestation many years before the diagnosis of a hematological disease, including the myeloproliferative neoplasms. Patients should be clinically followed and all transient or provoking risk factors avoided.

In younger patients with unprovoked VTE genetic thrombophilia testing should be considered (Table 6). This practical personalized approach can be summarized as follow:

- > 60 years: no genetic thrombophilia screening (Table 7)
- 50–60 years: consider what risk factors for VTE are present (Tables 1 and 2) and—if possible—avoid
- < 50 years: consider if thromboembolism (TE) may be the first cancer manifestation, including hematologic and myeloproliferative neoplasm and PNH
- < 50 years, female patients: consider as well autoimmune disease (i.e. Lupus Anticoagulans, antiphospholipid syndrome, Table 2)
- < 50 years, male, idiopathic: perform genetic thrombophilia screening
- < 40 years: consider illicit drug consumption or therapy (including cocaine, testosterone, erythropoietin)
- < 40 years female: exclude minor transient risk factors (Table 2). If TE an unusual site (abdominal or upper limb): consider PNH, cancer, autoimmune disease (Table 2)
- < 40 years: if TE idiopathic, in unusual sites or paradoxical perform thrombophilia screening including genetic tests (Table 6)
- < 40 years, female: if TE during pregnancy contraceptive or hormonal replacement or TE before prescribing hormonal replacement or multiple inexplicable pregnancy losses: perform genetic thrombophilia screening (Table 6)

**Table 6:** Proposed indications for venous thrombophilia screening

Idiopathic VTE < 50 years
Young patients with arterial ischemia caused by paradoxical embolism (right-to-left shunt)
VTE in unusual sites
Women with VTE during pregnancy or puerperium
Women with VTE during use of oral contraceptive or hormonal replacement
Women with VTE before prescribing hormonal replacement
Women with multiple inexplicable pregnancy losses
Young women with a strongly positive family history, before prescribing oral contraceptive
First VTE and a positive family history for VTE

Adapted from [18] and [19]

**Table 7:** Situations when a thrombophilia screening should not be performed

Young women with a negative personal and familial history, before prescribing oral contraceptive
Patient with tumor (active or inactive)
Patient with VTE after surgery and/or trauma
Patient > 60 years
Patient > 50 years with 1 or multiple strong risk factors
Relative 1. or 2. grades with VTE > 60 years
Patient without descendents or 1st degree relatives
Thrombophilia screening should not be performed in the acute phase after VTE diagnosis

### Genetic thrombophilia testing

Genetic thrombophilia screening—indicated only in selected patients [49]—include inherited deficiency of the natural anticoagulants protein C, protein S, antithrombin, and the mutation of factor V Leiden and the prothrombin gene. Blood group, hyperhomocysteinemia and dysfibrinogenemia are other inherited causes of thrombophilia (Table 4). In the search for a possible hereditary thrombophilia we recommend adherence to the above-mentioned main defects. The heterozygous or homozygous mutation of methylenetetrahydrofolate reductase (MTHFR)- 677C → T, which in the past was considered, has not been confirmed as a risk factor for the first VTE or for relapse (either alone or in combination with the F V Leiden [50, 51]) and its determination is redundant [52].

A genetic predisposition to VTE is supposed in the following cases: (1) in patients with VTE before 40 years of age, (2) VTE at a young age—before 50 years—with a weak risk factor, (3) considerably positive family history of VTE in two generations, (4) VTE in an unusual site.

Table 6 highlights the proposed indications for a venous thrombophilia screening. Thrombophilia screening is also important in order to estimate the risk of recurrence according to baseline risk factor profiles by detection of acquired thrombophilia. Major risk factors like malignancy or antiphospholipid syndrome influences the thrombosis risk and have clinical consequences regarding the anticoagulation therapy.

In conclusion, a clinical thrombophilia evaluation is indicated in all patients with VTE. According to age, acquired predisposing risk factors, duration of therapy and the patient’s requirement the physician determines the clinical likelihood and the indication—or not—to search for an inherited thrombophilia.

## Avoid universal screening

Universal screening of the general population to assess the venous thrombosis risk is unjustifiable [53], expensive and should be avoided [54, 55]. Consensus exists that universal unselected screening of young women before prescribing estrogen containing oral contraceptives is clearly not indicated [56]. The low prevalence in the general population of thrombophilia genetic defects requires a high number of patients to find out a mutation and even more to avoid one VTE. A source of trouble in clinical practice is the incorrect prescription of the screening test: some tests are frequently omitted and a negative thrombophilia screening may give a false hope. We strongly suggest to avoid thrombophilia screening in this scenario, especially true partial screening, because it is clinically futile and expensive [57]. First, we advise to assess the personal and patient’s family history of VTE. If a relative has had a VTE, it is important to ask at what age and in what context. A strong family history of VTE and/or relatives with VTE before 50 years make inherited thrombophilia more likely and laboratory testing should be certainly considered. We advise, whenever possible, to screen the Index patient. In all other cases thrombophilia screening is not indicated (Table 7).

## Selected screening

Knowledge of inherited thrombophilia may be important for young patients to avoid supplementary risk factors for VTE. Particularly smoking, dehydration, immobilization, estrogen containing medication (combined oral contraceptives or postmenopausal hormone replacement) should be avoided [37]. If chemotherapy is necessary, the treating physician should evaluate drug prophylaxis against VTE. After stopping anticoagulant therapy, in risk situations like pregnancy, acute illness, surgery, immobilization or long flights over 4 h a thromboembolic prophylaxis should be evaluated and respectively given [20]. Knowledge of the relative risk of possible pregnancy complications associated with the thrombophilic trait help in the clinical decision and management [58]. Particularly, a prophylaxis with low molecular weight heparin, Aspirin, substitution of coagulation factors and/or other management procedures should be implemented according to anamnesis, genetic defect and thromboembolic risk [59]. For each patient assessment of the individual risk during pregnancy and puerperium and a comprehensive evaluation of potential pregnancy complications depending on the genetic defect may help in the clinical decision [60]. Moreover, diagnosis of antiphospholipid syndrome either isolated or associated to other autoimmune disease like systemic lupus erythematosus (SLE) in females of childbearing age is important to evaluate preeclampsia, fetal loss, and preterm birth that are well-known risks in such pregnancies [61]. Women with antiphospholipid antibodies or antiphospholipid syndrome and lupus nephritis represent a group with high risk for obstetric complications. Factors such as appropriate preconception counseling, medication adjustment, strict disease control prior to pregnancy, and intensive surveillance during and after pregnancy are essential to improve pregnancy outcome and ensure the best maternal and fetal prognosis.

## Family screening

Detection of an inherited genetic defect induce a physician’s reflection about family screening. Thrombophilia screening in relatives should be selective and performed after adolescence or before prescribing birth control pills, especially to young Index patient’s daughters. All other young family female members should be additionally evaluated for possible screening. The relatives should be informed that a genetic mutation is only one risk factor predisposing to venous thromboembolic disease. Not all patients with the same mutation will develop a thrombosis or PE or will become ill.

Index patient’s relatives undergo a targeted thrombophilia screening, i.e. just the detected defect should be ruled out. Degree of relationship, age, therapy, indication or contraindication for anticoagulation should be included in the evaluation before performing the screening. As indicated above, potential risk of false positive results and over-treatment should be kept as low as possible. Therefore, vitamin K-deficiency in young women, drugs with potential interactions with the tests, circadian variation of the coagulation factors should all be considered in the final evaluation [62]. All results of functional tests outside the normal reference ranges and suspect as a deficiency should be confirmed in a second blood sampling, at the earliest after one month.

First degree relatives should be informed—concerning the detected defect—about the relative increased risk of first VTE, transient additional risk factors, possible thromboembolic prophylaxis (Table 5). Evaluation of VTE risk should be regularly repeated and adapted based on age, on new risk factors or illness. Members of families with strong positive VTE’s history but negative thrombophilia screening require comprehensive information. Thus, transient risk factors should be avoided whenever possible and women should receive an alternative medication to estrogen-containing pills or devices.

## Role and consequences of thrombophilia screening:

### Regarding thrombotic risk

Goals of thrombophilia screening are the search for possible causes of thrombosis [37, [63] and the identification of the patients who could benefit from a continuous anticoagulation after the first event. Other aims of the investigation are the identification of family members, at which VTE can be prevented by avoiding risk factors and/or with drug prophylaxis, as well as the counseling of patients, relatives and supervising physicians. The following parameters are helpful in the estimation of the individual recurrence risk for VTE and for the decision of the duration of anticoagulation [64]:

1. The circumstances of the VTE: postoperatively (cumulative recurrence risk after 5 years: approx. 3%), versus non-surgical risk factors, e.g. estrogenic hormone preparations, pregnancy, leg injury, flights over 4 h, plaster cast, 5–12 weeks postoperatively (cumulative risk of recurrence after 5 years: approx. 15%) versus idiopathic (cumulative recurrence risk after 5 years: approx. 30%) [65].
2. The location of the VTE: distal versus proximal.
3. The number of recurrent VTE events: recurrent VTE have an increased risk of recurrence (1.5) compared to first VTE [66].
4. The age > 60 years [67]; the sex (male gender: risk of recurrence 1.6 [68]); the body mass index: BMI 26–30 kg/m2: risk of recurrence approx. 2, BMI > 30 kg/m2: risk of recurrence approx. 5 [69].
5. The antiphospholipid antibodies: medium to high titer anticardiolipin antibody type IgG: risk of recurrence approx. 2; Lupus anticoagulant: risk of recurrence 6–8 [70].
6. Hereditary thrombophilia: antithrombin deficiency: risk of relapse approx. 2 [71]; homozygous factor V Leiden mutation risk of recurrence: approx. 2–3 [72]; Compound heterozygous factor V Leiden and Prothrombin gene G20210A mutation: risk of relapse approx. 2–5 [73].
7. D-Dimer level after anticoagulation discontinuation: normal D-Dimer: risk of recurrence: approx. 1.7 (males) and 0.4 (females), high D-Dimer (> 700 ng/ml) approx. 8.0 with males > females [74–76].
8. Persistent high FVIII:C > 90th percentile of the patient population (> 234%): risk of recurrence ca. 6 [77].
9. All diseases with high thrombotic risk (Table 2).

### Regarding therapy of thrombosis

The thrombophilia investigation should not be performed with the individual goal of defining the duration of the anticoagulation therapy. In the comprehensive assessment and advice, in addition to the risk of recurrence, the risk of bleeding under anticoagulation therapy should be estimated and the individual preference of the patient included.

The evidence shows a subordinate role of thrombophilia in the prediction of a recurrence of thrombosis. Three prognostic models for individual VTE recurrence risk after discontinuation of anticoagulation after one idiopathic VTE-HERDOO2 score, “Vienna prediction model” and DASH score—do not take thrombophilia into account [78]. In the score of Franco Moreno et al. [79] the genetic thrombophilia is statistically significant, but only as a retrospective observation, no prospective validation was performed with this score. If evidence of hereditary thrombophilia is present with possible consequences for offsprings, especially women of childbearing age, a family status (targeted partial thrombophilia evaluation) is recommended. It helps in the decision in risk situations for preventive action only by means of conservative measures (e.g. compression stockings, hydration, abstinence from estrogens) or even to apply thromboprophylaxis with medication. In addition, hereditary thrombophilia is an important component of thrombotic risk scores, such as “Caprini” and “Rodgers” for the risk stratification of patients within the context of perioperative thromboprophylaxis [80].

## Role of a positive diagnosis of thrombophilia regarding thrombosis prophylaxis

If a thrombophilic variance is detected in a family member, the question arises how to proceed concerning prophylaxis. The first risk stratification of patients is based on the personal history. In addition, physical habitus, age, type of coagulation anomaly, type of mutation (heterozygous or homozygous), underlying disease and other risk factors should be taken into consideration. In these patients, we generally recommend to avoid immobilization and/or dehydration. Especially with varicose compression stockings in risk situations (immobilization, travel longer than 4 h, pregnancy, postoperatively) are recommended. In these risk situations we recommend a careful drug prophylaxis and in women we favor against the prescription of estrogen-containing preparations (Table 8).

**Table 8:** Risk factors for VTE and preventive measures

Transient risk factors	
Immobilization	Compression stocking, LMWH prophylaxis, DOACs
Flight > 4 h	Regular movement during the flight, fluid intake, compression stockings, LMWH, DOACs
Surgery	Pneumatic and compression stockings, early mobilization, hydration, LMWH, DOACs
Pregnancy—Puerperium	Compression stockings, hydration, LMWH
Medication	Avoid estrogens, EPO, testosterone
Extended varicosis	Evaluation of surgical repair, stockings, LMWH
Smoking	Avoid or interrupt smoking

Persistent risk factors	
Myeloproliferative Neoplasm: PV, ET, PMF	Hematocrit and blood cells under therapeutic limits, prophylaxis or anticoagulation

*EPO* erythropoietin; *LMWH* low molecular weight heparin, dose adapted primarily to personal history, VTE risk, patient’s weight, renal function; *DOACs* direct oral anticoagulants; *PV* polycythaemia vera; *ET* essential thrombocythaemia; *PMF* primary myelofibrosis

## Role of a negative diagnosis of thrombophilia regarding thrombosis prophylaxis

If the family history is clearly strong positive and no laboratory thrombophilia is proved, the prophylactic measures are recommended as above. Even more, if the personal medical history is also positive for VTE.

## Role of thrombophilia in the context of a pregnancy

The physiological adaptations of the body, the blood circulation and the coagulation during pregnancy increase the risk of thrombosis. Although the risk is influenced ante-partum especially by the BMI, age, number of births, varicosis and post-partum due to premature birth, cesarean section and hemorrhage, the VTE risk in women with hereditary thrombophilia and positive family history is especially high. Possible obstetric complications in the presence of thrombophilic defects are e.g. pre-eclampsia in antithrombin- or protein S deficiency or the fetal growth retardation in factor V Leiden and prothrombin gene G20210A mutation [81]. The assessment of the personal risk prior to the initiation of medication for thromboembolic event prophylaxis and regular checks during pregnancy are indicated for these patients. Start (24th week of gestation or earlier), dose and duration of prophylaxis are individual to decide. We recommend the prophylaxis with low molecular weight Heparins (LMWH), risk and weight-adapted (usually 75–100 IU/kg body weight/day), until the onset of labor pains. An interval of 12 h from the last LMWH low-dose is enough to carry out a spinal anesthesia. An interdisciplinary management of these patients with involvement of gynecologists, midwives, anesthesiologists and hematologists during pregnancy, at delivery and in the post-partum period is strongly recommended.

## Future diagnostic perspectives

High-throughput sequencing methodology is now available and affordable for every-day genetics [82]. In the era of the GWAS it is possible, that genetic cohort analysis of pre-specified patients or healthy persons can reveal single polymorphisms, which alone or in combination are associated with thrombotic risk. Genetic risk scores or clustered panels of thrombotic genes with respect to this have already been published [34, 83, 84]. The benefit of such a wide genetic analysis, though, remains uncertain. Many of such gene candidates and their biological influence are at present unknown. The identification of variants of unknown significance (VUS) can be at present disturbing than helping. Moreover, the identification of variants, known to be associated to other diseases than thrombosis, such as the RUNX1 variation for leukemia or the aneuploidies can cause more problems than answer questions [84]. Should they be reported within the context of thrombophilia investigation or should they be silenced? Is there a risk for misinterpretation of the genetic results? How can we risk-stratify a VUS? These questions are still open, there is an urgent need for re-defining the indications and dimensions of extensive genetic testing.

## References

[1] Martinelli I, Bucciarelli P, Mannucci PM (2010). Thrombotic risk factors: basic pathophysiology. Crit Care Med.

[2] Lippi G, Franchini M (2008). Pathogenesis of venous thromboembolism: when the cup runneth over. Semin Thromb Hemost.

[3] Bagot CN, Arya R (2008). Virchow and his triad: a question of attribution. Br J Haematol.

[4] Stricker H, Colucci G, Godio M, Mossi G, Mombelli G (2003). The influence of a prolonged sitting position on the biochemical markers of coagulation activation in healthy subjects: evidence of reduced thrombin generation. J Thromb Haemost.

[5] Stricker H, Colucci G, Alberio L, Mombelli G (2006). Variation in coagulation inhibitors during prolonged sitting: possible pathogenetic mechanisms for travel-associated thrombosis. J Thromb Haemost.

[6] Colucci G, Stricker H, Roggiani W, Haeberli A, Mombelli G (2004). Venous stasis and thrombin generation. J Thromb Haemost.

[7] Singh S, Houng AK, Reed GL (2019). Venous stasis-induced fibrinolysis prevents thrombosis in mice: role of alpha2-antiplasmin. Blood.

[8] Ruhl H, Muller J, Waschenbach J, Oldenburg J, Dewald O, Potzsch B (2014). Short-term venous stasis induces fibrinolytic activation but not thrombin formation. J Atheroscler Thromb.

[9] Conway EM (2015). Reincarnation of ancient links between coagulation and complement. J Thromb Haemost.

[10] Oikonomopoulou K, Ricklin D, Ward PA, Lambris JD (2012). Interactions between coagulation and complement–their role in inflammation. Semin Immunopathol.

[11] Anderson FA, Wheeler HB, Goldberg RJ, Hosmer DW, Patwardhan NA, Jovanovic B, Forcier A, Dalen JE (1991). A population-based perspective of the hospital incidence and case-fatality rates of deep vein thrombosis and pulmonary embolism. The Worcester DVT Study. Arch Intern Med.

[12] Rosendaal FR (1999). Venous thrombosis: a multicausal disease. Lancet.

[13] Morange PE, Suchon P, Tregouet DA (2015). Genetics of venous thrombosis: update in 2015. Thromb Haemost.

[14] Cohn DM, Middeldorp S (2008). Early termination of the multicentre randomised clinical trial to evaluate the benefit of testing for thrombophilia following a first venous thromboembolism: the NOSTRADAMUS study. Ned Tijdschr Geneeskd.

[15] Middeldorp S (2011). Is thrombophilia testing useful?. Hematology Am Soc Hematol Educ Program.

[16] Cohn DM, Vansenne F, de Borgie CA, Middeldorp S (2012). Thrombophilia testing for prevention of recurrent venous thromboembolism. Cochrane Database Syst Rev.

[17] Middeldorp S (2016). Inherited thrombophilia: a double-edged sword. Hematology Am Soc Hematol Educ Program.

[18] Stevens SM, Woller SC, Bauer KA, Kasthuri R, Cushman M, Streiff M, Lim W, Douketis JD (2016). Guidance for the evaluation and treatment of hereditary and acquired thrombophilia. J Thromb Thrombolysis.

[19] Pernod G, Biron-Andreani C, Morange PE, Boehlen F, Constans J, Couturaud F, Drouet L, Jude B, Lecompte T, Le Gal G, Trillot N, Wahl D, French group on h, thrombosis, French Society of vascular m (2009). Recommendations on testing for thrombophilia in venous thromboembolic disease: a French consensus guideline. J Mal Vasc.

[20] Kearon C, Akl EA, Ornelas J, Blaivas A, Jimenez D, Bounameaux H, Huisman M, King CS, Morris TA, Sood N, Stevens SM, Vintch JRE, Wells P, Woller SC, Moores L (2016). Antithrombotic therapy for VTE disease: CHEST guideline and expert panel report. Chest.

[21] Egeberg O (1965). Inherited antithrombin deficiency causing thrombophilia. Thromb Diath Haemorrh.

[22] Griffin JH, Evatt B, Zimmerman TS, Kleiss AJ, Wideman C (1981). Deficiency of protein C in congenital thrombotic disease. J Clin Invest.

[23] Comp PC, Esmon CT (1984). Recurrent venous thromboembolism in patients with a partial deficiency of protein S. N Engl J Med.

[24] Uitte de Willige S, de Visser MC, Houwing-Duistermaat JJ, Rosendaal FR, Vos HL, Bertina RM (2005). Genetic variation in the fibrinogen gamma gene increases the risk for deep venous thrombosis by reducing plasma fibrinogen gamma’ levels. Blood.

[25] den Heijer M, Koster T, Blom HJ, Bos GM, Briet E, Reitsma PH, Vandenbroucke JP, Rosendaal FR (1996). Hyperhomocysteinemia as a risk factor for deep-vein thrombosis. N Engl J Med.

[26] Poort SR, Rosendaal FR, Reitsma PH, Bertina RM (1996). A common genetic variation in the 3'-untranslated region of the prothrombin gene is associated with elevated plasma prothrombin levels and an increase in venous thrombosis. Blood.

[27] Bertina RM, Koeleman BP, Koster T, Rosendaal FR, Dirven RJ, de Ronde H, van der Velden PA, Reitsma PH (1994). Mutation in blood coagulation factor V associated with resistance to activated protein C. Nature.

[28] Morange PE, Tregouet DA (2013). Current knowledge on the genetics of incident venous thrombosis. J Thromb Haemost.

[29] Tirado I, Mateo J, Soria JM, Oliver A, Martinez-Sanchez E, Vallve C, Borrell M, Urrutia T, Fontcuberta J (2005). The ABO blood group genotype and factor VIII levels as independent risk factors for venous thromboembolism. Thromb Haemost.

[30] Procare GG (2006). ABO blood group but not haemostasis genetic polymorphisms significantly influence thrombotic risk: a study of 180 homozygotes for the factor V Leiden mutation. Br J Haematol.

[31] Dentali F, Sironi AP, Ageno W, Turato S, Bonfanti C, Frattini F, Crestani S, Franchini M (2012). Non-O blood type is the commonest genetic risk factor for VTE: results from a meta-analysis of the literature. Semin Thromb Hemost.

[32] Germain M, Chasman DI, de Haan H, Tang W, Lindstrom S, Weng LC, de Andrade M, de Visser MC, Wiggins KL, Suchon P, Saut N, Smadja DM, Le Gal G, van Hylckama VA, Di Narzo A, Hao K, Nelson CP, Rocanin-Arjo A, Folkersen L, Monajemi R, Rose LM, Brody JA, Slagboom E, Aissi D, Gagnon F, Deleuze JF, Deloukas P, Tzourio C, Dartigues JF, Berr C, Taylor KD, Civelek M, Eriksson P, Cardiogenics C, Psaty BM, Houwing-Duitermaat J, Goodall AH, Cambien F, Kraft P, Amouyel P, Samani NJ, Basu S, Ridker PM, Rosendaal FR, Kabrhel C, Folsom AR, Heit J, Reitsma PH, Tregouet DA, Smith NL, Morange PE (2015). Meta-analysis of 65,734 individuals identifies TSPAN15 and SLC44A2 as two susceptibility loci for venous thromboembolism. Am J Hum Genet.

[33] Tregouet DA, Morange PE (2018). What is currently known about the genetics of venous thromboembolism at the dawn of next generation sequencing technologies. Br J Haematol.

[34] Downes K, Megy K, Duarte D, Vries M, Gebhart J, Hofer S, Shamardina O, Deevi SVV, Stephens J, Mapeta R, Tuna S, Al Hasso N, Besser MW, Cooper N, Daugherty L, Gleadall N, Greene D, Haimel M, Martin H, Papadia S, Revel-Vilk S, Sivapalaratnam S, Symington E, Thomas W, Thys C, Tolios A, Penkett CJ, BioResource N, Ouwehand WH, Abbs S, Laffan MA, Turro E, Simeoni I, Mumford AD, Henskens YMC, Pabinger I, Gomez K, Freson K (2019). Diagnostic high-throughput sequencing of 2396 patients with bleeding, thrombotic, and platelet disorders. Blood.

[35] Rees DC, Cox M, Clegg JB (1995). World distribution of factor V Leiden. Lancet.

[36] Rosendaal FR, Doggen CJ, Zivelin A, Arruda VR, Aiach M, Siscovick DS, Hillarp A, Watzke HH, Bernardi F, Cumming AM, Preston FE, Reitsma PH (1998). Geographic distribution of the 20210 G to A prothrombin variant. Thromb Haemost.

[37] Anderson FA, Spencer FA (2003). Risk factors for venous thromboembolism. Circulation.

[38] Vossen CY, Walker ID, Svensson P, Souto JC, Scharrer I, Preston FE, Palareti G, Pabinger I, van der Meer FJ, Makris M, Fontcuberta J, Conard J, Rosendaal FR (2005). Recurrence rate after a first venous thrombosis in patients with familial thrombophilia. Arterioscler Thromb Vasc Biol.

[39] Kenet G, Nowak-Gottl U (2012). Venous thromboembolism in neonates and children. Best Pract Res Clin Haematol.

[40] Parker RI (2010). Thrombosis in the pediatric population. Crit Care Med.

[41] Young G, Albisetti M, Bonduel M, Brandao L, Chan A, Friedrichs F, Goldenberg NA, Grabowski E, Heller C, Journeycake J, Kenet G, Krumpel A, Kurnik K, Lubetsky A, Male C, Manco-Johnson M, Mathew P, Monagle P, van Ommen H, Simioni P, Svirin P, Tormene D, Nowak-Gottl U (2008). Impact of inherited thrombophilia on venous thromboembolism in children: a systematic review and meta-analysis of observational studies. Circulation.

[42] Holzhauer S, Goldenberg NA, Junker R, Heller C, Stoll M, Manner D, Mesters R, Krumpel A, Stach M, Nowak-Gottl U (2012). Inherited thrombophilia in children with venous thromboembolism and the familial risk of thromboembolism: an observational study. Blood.

[43] Nowak-Gottl U, van Ommen H, Kenet G (2018). Thrombophilia testing in children: what and when should be tested?. Thromb Res.

[44] Varga EA, Kujovich JL (2012). Management of inherited thrombophilia: guide for genetics professionals. Clin Genet.

[45] Goldberg RJ, Seneff M, Gore JM, Anderson FA, Greene HL, Wheeler HB, Dalen JE (1987). Occult malignant neoplasm in patients with deep venous thrrombosis. Arch Intern Med.

[46] Prandoni P, Lensing AW, Buller HR, Cogo A, Prins MH, Cattelan AM, Cuppini S, Noventa F, ten Cate JW (1992). Deep-vein thrombosis and the incidence of subsequent symptomatic cancer. N Engl J Med.

[47] Delluc A, Antic D, Lecumberri R, Ay C, Meyer G, Carrier M (2017). Occult cancer screening in patients with venous thromboembolism: guidance from the SSC of the ISTH. J Thromb Haemost.

[48] Carrier M, Lazo-Langner A, Shivakumar S, Tagalakis V, Zarychanski R, Solymoss S, Routhier N, Douketis J, Danovitch K, Lee AY, Le Gal G, Wells PS, Corsi DJ, Ramsay T, Coyle D, Chagnon I, Kassam Z, Tao H, Rodger MA, Investigators S (2015). Screening for Occult cancer in unprovoked venous thromboembolism. N Engl J Med.

[49] Colucci G, Tsakiris DA (2017). Thrombophilia screening: universal, selected, or neither?. Clin Appl Thromb Hemost.

[50] Ocal IT, Sadeghi A, Press RD (1997). Risk of venous thrombosis in carriers of a common mutation in the homocysteine regulatory enzyme methylenetetrahydrofolate reductase. Mol Diagn.

[51] Simone B, De Stefano V, Leoncini E, Zacho J, Martinelli I, Emmerich J, Rossi E, Folsom AR, Almawi WY, Scarabin PY, den Heijer M, Cushman M, Penco S, Vaya A, Angchaisuksiri P, Okumus G, Gemmati D, Cima S, Akar N, Oguzulgen KI, Ducros V, Lichy C, Fernandez-Miranda C, Szczeklik A, Nieto JA, Torres JD, Le Cam-Duchez V, Ivanov P, Cantu-Brito C, Shmeleva VM, Stegnar M, Ogunyemi D, Eid SS, Nicolotti N, De Feo E, Ricciardi W, Boccia S (2013). Risk of venous thromboembolism associated with single and combined effects of factor V Leiden, prothrombin 20210A and methylenetethraydrofolate reductase C677T: a meta-analysis involving over 11,000 cases and 21,000 controls. Eur J Epidemiol.

[52] Connors JM (2017). Thrombophilia testing and venous thrombosis. N Engl J Med.

[53] Simpson EL, Stevenson MD, Rawdin A, Papaioannou D (2009). Thrombophilia testing in people with venous thromboembolism: systematic review and cost-effectiveness analysis. Health Technol Assess.

[54] Wu O, Robertson L, Twaddle S, Lowe G, Clark P, Walker I, Brenkel I, Greaves M, Langhorne P, Regan L, Greer I, Thrombosis R, Economic Assessment of Thrombophilia Screening S (2005). Screening for thrombophilia in high-risk situations: a meta-analysis and cost-effectiveness analysis. Br J Haematol.

[55] Wu O, Robertson L, Twaddle S, Lowe GD, Clark P, Greaves M, Walker ID, Langhorne P, Brenkel I, Regan L, Greer I (2006). Screening for thrombophilia in high-risk situations: systematic review and cost-effectiveness analysis. The thrombosis: risk and economic assessment of thrombophilia screening (TREATS) study. Health Technol Assess.

[56] Creinin MD, Lisman R, Strickler RC (1999). Screening for factor V Leiden mutation before prescribing combination oral contraceptives. Fertil Steril.

[57] Favaloro EJ (2014). The futility of thrombophilia testing. Clin Chem Lab Med.

[58] Ormesher L, Simcox LE, Tower C, Greer IA (2017). ‘To test or not to test’, the arguments for and against thrombophilia testing in obstetrics. Obstet Med.

[59] Croles FN, Nasserinejad K, Duvekot JJ, Kruip MJ, Meijer K, Leebeek FW (2017). Pregnancy, thrombophilia, and the risk of a first venous thrombosis: systematic review and bayesian meta-analysis. BMJ.

[60] Giovanni L, Maria LG, Mauro R, Carlotta M, Federica R, Fabrizio P, Sheba J, Giuseppe DP, Alessandro B, Elio C, Herbert V (2011). Thrombophilia and damage of kidney during pregnancy. J Prenat Med.

[61] Tektonidou MG, Andreoli L, Limper M, Amoura Z, Cervera R, Costedoat-Chalumeau N, Cuadrado MJ, Dorner T, Ferrer-Oliveras R, Hambly K, Khamashta MA, King J, Marchiori F, Meroni PL, Mosca M, Pengo V, Raio L, Ruiz-Irastorza G, Shoenfeld Y, Stojanovich L, Svenungsson E, Wahl D, Tincani A, Ward MM (2019). EULAR recommendations for the management of antiphospholipid syndrome in adults. Ann Rheum Dis.

[62] Tripodi A (2012). Problems and solutions for testing hemostasis assays while patients are on anticoagulants. Semin Thromb Hemost.

[63] Ortel TL (2010). Acquired thrombotic risk factors in the critical care setting. Crit Care Med.

[64] Kearon C (2004). Long-term management of patients after venous thromboembolism. Circulation.

[65] Prandoni P, Noventa F, Ghirarduzzi A, Pengo V, Bernardi E, Pesavento R, Iotti M, Tormene D, Simioni P, Pagnan A (2007). The risk of recurrent venous thromboembolism after discontinuing anticoagulation in patients with acute proximal deep vein thrombosis or pulmonary embolism. A prospective cohort study in 1,626 patients. Haematologica.

[66] Baglin T, Luddington R, Brown K, Baglin C (2003). Incidence of recurrent venous thromboembolism in relation to clinical and thrombophilic risk factors: prospective cohort study. Lancet.

[67] White RH (2003). The epidemiology of venous thromboembolism. Circulation.

[68] McRae S, Tran H, Schulman S, Ginsberg J, Kearon C (2006). Effect of patient's sex on risk of recurrent venous thromboembolism: a meta-analysis. Lancet.

[69] Heit JA, Mohr DN, Silverstein MD, Petterson TM, O'Fallon WM, Melton LJ (2000). Predictors of recurrence after deep vein thrombosis and pulmonary embolism: a population-based cohort study. Arch Intern Med.

[70] Galli M, Luciani D, Bertolini G, Barbui T (2003). Lupus anticoagulants are stronger risk factors for thrombosis than anticardiolipin antibodies in the antiphospholipid syndrome: a systematic review of the literature. Blood.

[71] De Stefano V, Simioni P, Rossi E, Tormene D, Za T, Pagnan A, Leone G (2006). The risk of recurrent venous thromboembolism in patients with inherited deficiency of natural anticoagulants antithrombin, protein C and protein S. Haematologica.

[72] Segal JB, Brotman DJ, Necochea AJ, Emadi A, Samal L, Wilson LM, Crim MT, Bass EB (2009). Predictive value of factor V Leiden and prothrombin G20210A in adults with venous thromboembolism and in family members of those with a mutation: a systematic review. JAMA.

[73] De Stefano V, Martinelli I, Mannucci PM, Paciaroni K, Chiusolo P, Casorelli I, Rossi E, Leone G (1999). The risk of recurrent deep venous thrombosis among heterozygous carriers of both factor V Leiden and the G20210A prothrombin mutation. N Engl J Med.

[74] Palareti G, Cosmi B, Legnani C, Tosetto A, Brusi C, Iorio A, Pengo V, Ghirarduzzi A, Pattacini C, Testa S, Lensing AW, Tripodi A, Investigators P (2006). D-dimer testing to determine the duration of anticoagulation therapy. N Engl J Med.

[75] Cosmi B, Legnani C, Tosetto A, Pengo V, Ghirarduzzi A, Testa S, Prisco D, Poli D, Tripodi A, Marongiu F, Palareti G, Investigators P (2010). Usefulness of repeated D-dimer testing after stopping anticoagulation for a first episode of unprovoked venous thromboembolism: the PROLONG II prospective study. Blood.

[76] Palareti G, Legnani C, Antonucci E, Cosmi B, Poli D, Testa S, Tosetto A, Ageno W, Falanga A, Ferrini PM, Pengo V, Prandoni P, Investigators D (2019). D-dimer testing, with gender-specific cutoff levels, is of value to assess the individual risk of venous thromboembolic recurrence in non-elderly patients of both genders: a post hoc analysis of the DULCIS study. Intern Emerg Med.

[77] Kyrle PA, Minar E, Hirschl M, Bialonczyk C, Stain M, Schneider B, Weltermann A, Speiser W, Lechner K, Eichinger S (2000). High plasma levels of factor VIII and the risk of recurrent venous thromboembolism. N Engl J Med.

[78] Ensor J, Riley RD, Moore D, Snell KI, Bayliss S, Fitzmaurice D (2016). Systematic review of prognostic models for recurrent venous thromboembolism (VTE) post-treatment of first unprovoked VTE. BMJ Open.

[79] Franco Moreno AI, Garcia Navarro MJ, Ortiz Sanchez J, Martin Diaz RM, Madronal Cerezo E, de Ancos Aracil CL, Cabello Clotet N, Perales Fraile I, Gimeno Garcia S, Montero Hernandez C, Zapatero Gaviria A, Ruiz Giardin JM (2016). A risk score for prediction of recurrence in patients with unprovoked venous thromboembolism (DAMOVES). Eur J Intern Med.

[80] Bahl V, Hu HM, Henke PK, Wakefield TW, Campbell DA, Caprini JA (2010). A validation study of a retrospective venous thromboembolism risk scoring method. Ann Surg.

[81] Larciprete G, Gioia S, Angelucci PA, Brosio F, Barbati G, Angelucci GP, Frigo MG, Baiocco F, Romanini ME, Arduini D, Cirese E (2007). Single inherited thrombophilias and adverse pregnancy outcomes. J Obstet Gynaecol Res.

[82] Turnbull C, Scott RH, Thomas E, Jones L, Murugaesu N, Pretty FB, Halai D, Baple E, Craig C, Hamblin A, Henderson S, Patch C, O'Neill A, Devereau A, Smith K, Martin AR, Sosinsky A, McDonagh EM, Sultana R, Mueller M, Smedley D, Toms A, Dinh L, Fowler T, Bale M, Hubbard T, Rendon A, Hill S, Caulfield MJ, Genomes P (2018). The 100 000 genomes project: bringing whole genome sequencing to the NHS. BMJ.

[83] Crous-Bou M, Harrington LB, Kabrhel C (2016). Environmental and genetic risk factors associated with venous thromboembolism. Semin Thromb Hemost.

[84] Crous-Bou M, De Vivo I, Camargo CA, Varraso R, Grodstein F, Jensen MK, Kraft P, Goldhaber SZ, Lindstrom S, Kabrhel C (2016). Interactions of established risk factors and a GWAS-based genetic risk score on the risk of venous thromboembolism. Thromb Haemost.

